# Supplementary material for: Identification of an early-heading mutant in Indonesian native rice cultivar: ‘Gemdjah Beton-10’
Source: Breed Sci. 2025 Nov 1;75(5):412–20. doi: 10.1270/jsbbs.25024 (PMC13129580; doi:10.1270/jsbbs.25024)
Supplement: Supplementary file 1 — Supplemental Figures [file 75_412_s1.pdf]

Nagalla et al./Breeding Science
doi: 10.1270/jsbbs.25024

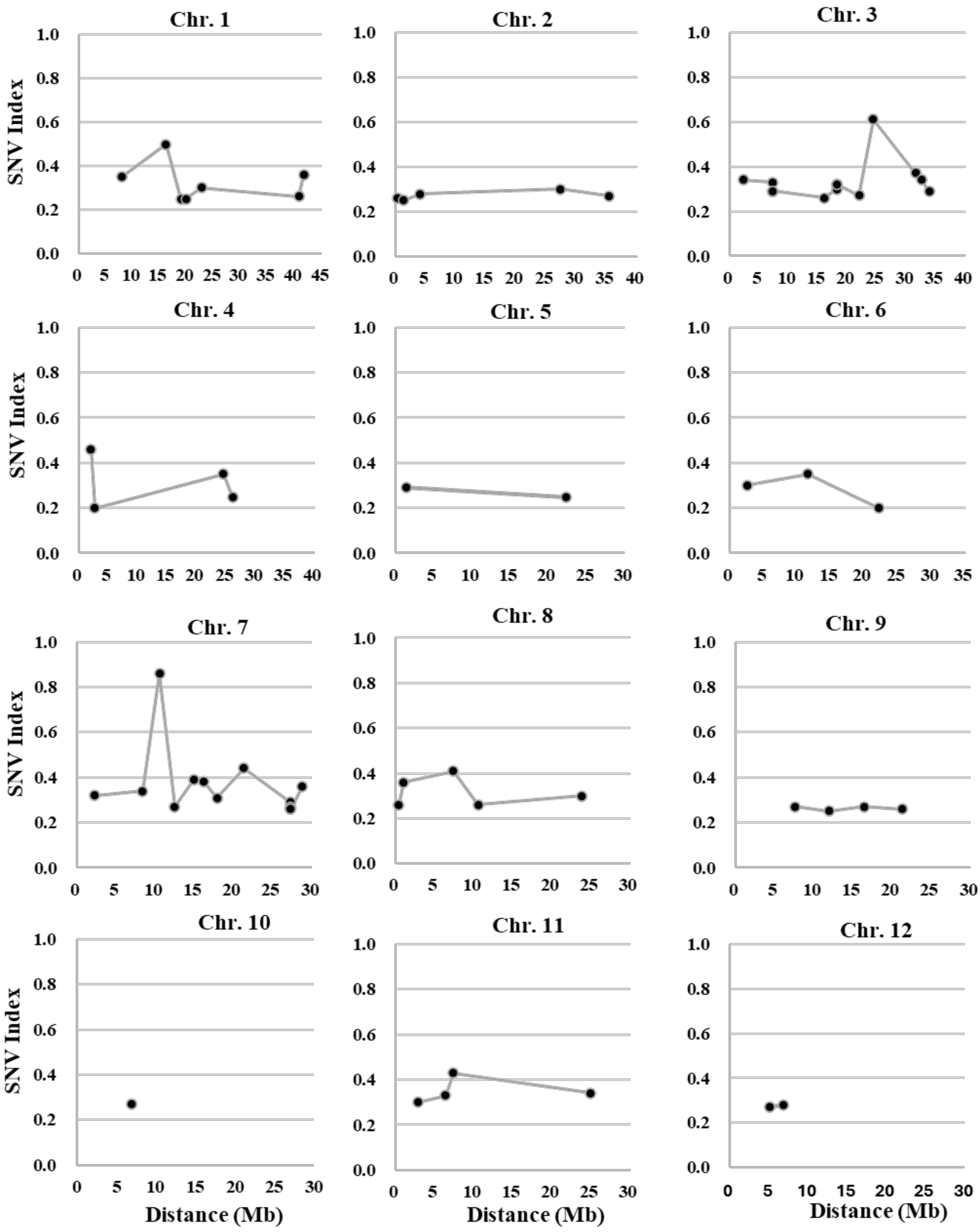

Supplemental Fig. 1.

SNV index plots showing the mutations in GB-10 for each chromosome (Chr).

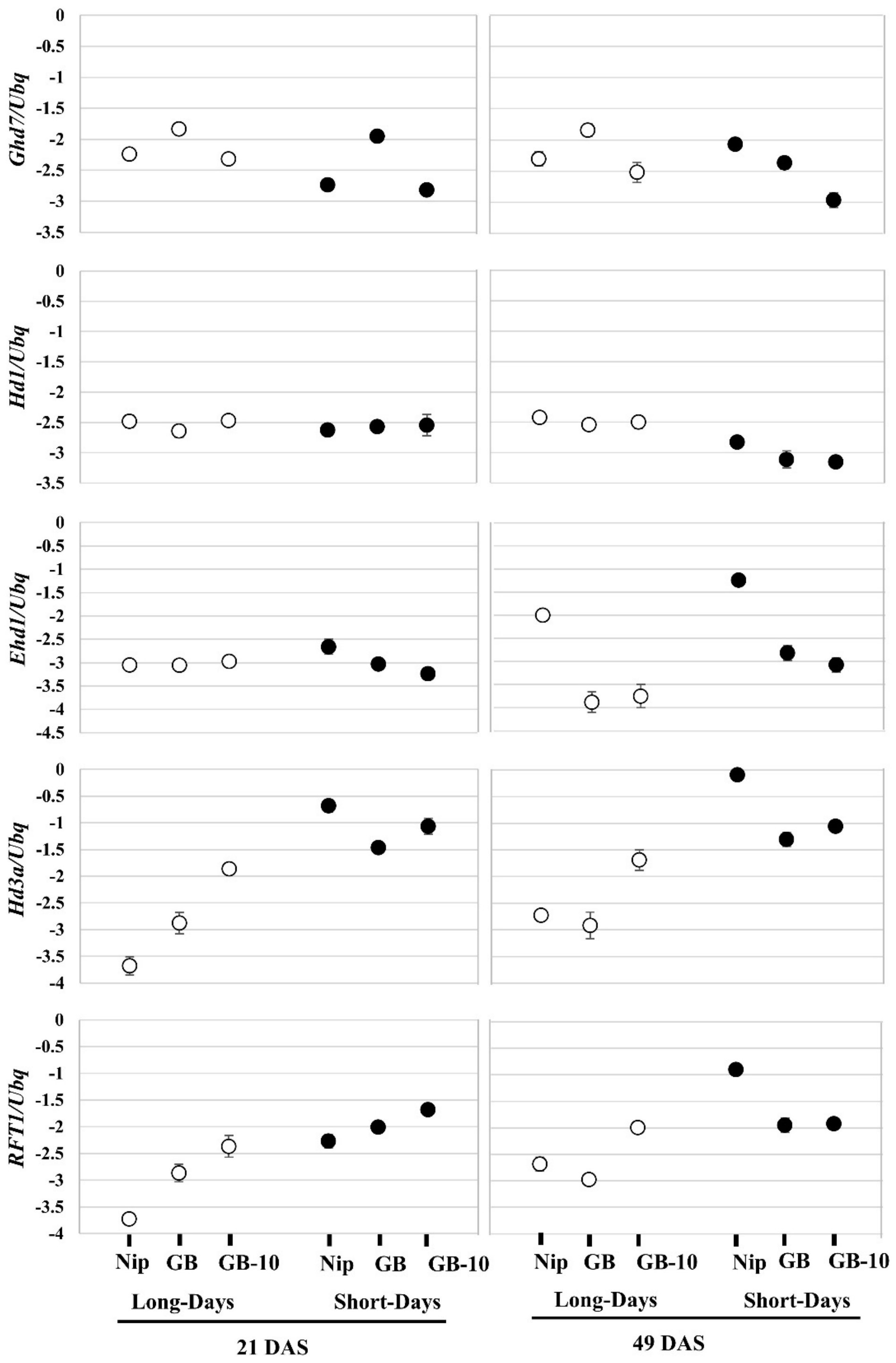

Supplemental Fig. 2.

Relative gene expression of *Ghd7*, *Hd1*, *Ehd1*, *Hd3a* and *RFT1* of the cv. Nipponbare, GB and GB-10 mutant under LD and SD conditions. 21days old (left) and 49 days old (right) leaf blade samples were harvested 2hr after dawn. The significance of the difference was assessed by Student's t-test (***P* < 0.01). The relative gene expression was shown in the logarithmic Y-axis.

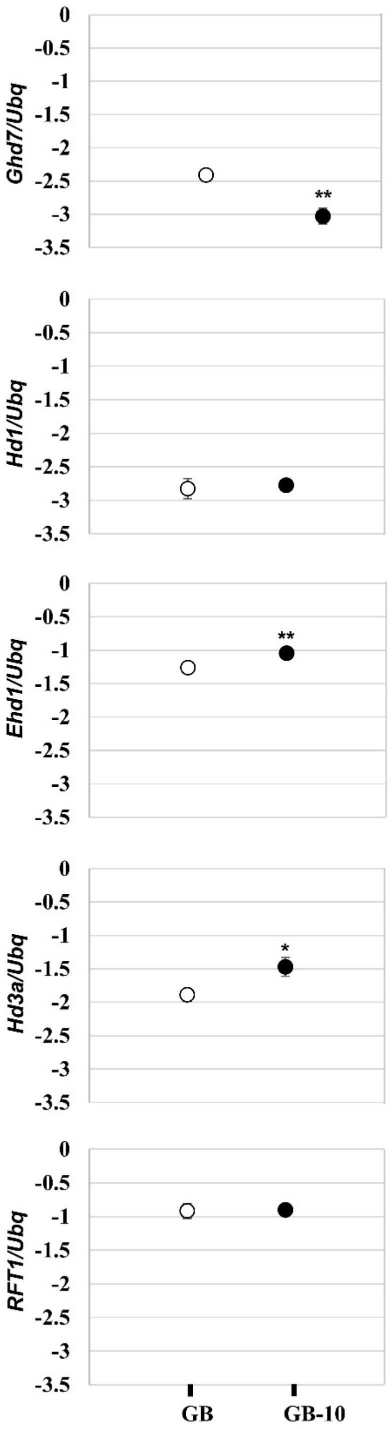

Supplemental Fig. 3.

Relative gene expression of *Ghd7*, *Hd1*, *Ehd1*, *Hd3a* and *RFT1* of the GB and GB-10 mutant under LD conditions. 90 DAS plant leaf blade samples were harvested 2hr after dawn. Data are means ± S. D. (n = 3 or 4 biological replicates). Relative gene expression was shown in the logarithmic Y-axis.
